# Integrated Single-Cell and Bulk RNA Sequencing Identifies Macrophage Heterogeneity and Mitophagy-Related Biomarkers in Idiopathic Pulmonary Fibrosis

**DOI:** 10.3390/ijms27104201

**Published:** 2026-05-08

**Authors:** Chen Shang, Gao Huang

**Affiliations:** School of Qihuang Medicine, Guizhou University of Traditional Chinese Medicine, Guiyang 550025, China; shangchen@stu.gzy.edu.cn

**Keywords:** idiopathic pulmonary fibrosis, macrophages, mitophagy, single-cell RNA sequencing, biomarkers

## Abstract

Mitophagy clears damaged mitochondria and maintains normal macrophage function. Clarifying the associations between idiopathic pulmonary fibrosis (IPF), macrophages, and mitophagy is crucial for early diagnosis and clinical management. Core macrophage subsets were identified as M2 macrophages via single-cell RNA sequencing and immune infiltration analysis. Differentially expressed genes related to this subset were obtained. Integrated differential expression analysis, weighted gene co-expression network analysis, machine learning, and expression verification were applied to screen biomarkers. *CD163* and *SPP1* were identified through biomarker screening, both showing significantly increased expression in IPF. Functional enrichment showed that these biomarkers are mainly involved in cell cycle checkpoints and ciliopathies. Immune microenvironment analysis identified 16 immune cell types with significant differences between IPF and control groups, among which T helper 2 cells were strongly positively correlated with *CD163*. A total of nine drugs were found to be associated with *CD163* and *SPP1*. The expression of these biomarkers changed dynamically during M2 macrophage differentiation. This study integrates single-cell and bulk transcriptomics analysis to reveal the critical roles of *CD163* and *SPP1* in the IPF macrophage–mitochondrial autophagy axis, a novel framework for understanding the macrophage–mitophagy axis in IPF pathogenesis.

## 1. Introduction

Idiopathic pulmonary fibrosis (IPF) constitutes a distinct clinical entity within the spectrum of interstitial lung diseases, characterized by relentless, progressive fibrotic replacement of lung parenchyma, culminating in inexorable respiratory decline and mortality [1]. Although it has a low incidence, it exhibits an extremely high mortality rate. The disease predominantly occurs in middle-aged and elderly populations with an age over 50 years, with clinical manifestations including persistent dry cough, exertional dyspnea, and progressive decline in pulmonary function, significantly impairing patients’ quality of life [2]. Although the etiology of IPF remains not completely understood, aging and genetic predisposition (e.g., telomere shortening, *MUC5B* promoter variants) are established risk factors [3]. Environmental triggers such as smoking and occupational exposures (e.g., silica, metal dust) may further exacerbate fibrotic progression via dysregulated epithelial–mesenchymal crosstalk [3,4]. The pathogenesis of IPF involves abnormal wound-healing responses, characterized by recurrent alveolar epithelial injury, dysregulated fibroblast activation, and excessive extracellular matrix (ECM) deposition, ultimately leading to a distorted lung architecture [5]. Current clinical management relies primarily on antifibrotic agents such as pirfenidone and nintedanib, which can slow the rate of pulmonary function decline but fail to reverse fibrotic progression. Patients face limited therapeutic options; the median survival period is 5.7 years [6,7]. Elucidating the pathogenic mechanisms of IPF and identifying effective biomarkers hold critical significance for early disease detection and the development of personalized treatment strategies.

Macrophages, as key effector cells of the innate immune system, play a central role in tissue homeostasis and injury repair, with the M1 phenotype contributing to tissue damage through the secretion of pro-inflammatory factors such as TNF-α and IL-1β, while the M2 phenotype promotes tissue repair and fibrosis via the release of IL-10 and TGF-β [8,9]. Ding et al. (2025) further confirmed that CD163+ macrophages are enriched in patients with rapid pulmonary fibrosis and mouse models, and they promote disease progression by secreting osteopontin (OPN), indicating that the CD163+ macrophage–OPN axis may be a potential therapeutic target for rapid pulmonary fibrosis—their research laid an important foundation for exploring the role of CD163+ macrophages in pulmonary fibrosis progression [10]. Mitophagy, a selective autophagy process that maintains cellular homeostasis by clearing damaged mitochondria, is closely linked to fibrotic diseases when dysregulated [11]. Unlike previous studies that focused on the role of CD163+ macrophages in pulmonary fibrosis, this study specifically focuses on IPF and takes mitophagy as an innovative entry point to explore the regulatory mechanism of the macrophage–mitophagy axis in IPF. In IPF, M1 macrophages exacerbate alveolar epithelial injury, whereas M2 macrophages promote fibrosis through TGF-β [8]. Concurrently, mitophagy deficiency leads to metabolic disturbances and cellular senescence, as demonstrated by tetrandrine’s ability to rescue mitochondrial dysfunction via the PINK1/Parkin pathway [12]. Research further indicates that the Akt1 signaling pathway promotes pulmonary fibrosis by inhibiting mitophagy and enhancing macrophage apoptosis resistance [13]. However, currently, research on IPF diagnosis and treatment targeted at macrophage polarization and mitophagy-related genes remains inadequate: there is a lack of integrated biomarkers combining macrophage polarization and mitophagy status and immature targeted therapeutic strategies, as well as a lack of understanding of the dynamic regulatory mechanisms of the macrophage–mitophagy axis. Therefore, future efforts necessitate in-depth exploration of the interaction mechanisms between these processes alongside the development of multi-omics biomarkers and targeted intervention strategies (such as modulation of the PINK1/Akt1 pathway) to provide new avenues for the accurate diagnosis and treatment of IPF.

Single-cell RNA sequencing (scRNA-seq) constitutes a transformative advancement of traditional bulk sequencing through resolving gene expression profiles at a single-cell resolution, enabling precise identification of cellular heterogeneity and characterization of rare functional subpopulations [14,15]. Studies have revealed significant phenotypic remodeling of distal lung epithelial cells during pulmonary fibrosis, identifying a novel pathological KRT5^−^/KRT17^+^ epithelial cell subpopulation. This cell population, exhibiting ECM-producing properties, is specifically enriched in fibrotic lung tissues [16]. In a study investigating IPF using scRNA-seq, a specific subpopulation of fibroblasts exhibiting high expression of ECM-related genes was identified. In this study, it was further proposed that close interactions between epithelial cells and fibroblasts, mediated through ligand–receptor pairs, may be critical to the pathogenesis of IPF [17]. Research using scRNA-seq reveals key cell populations during the development of IPF, providing insights into its pathogenic mechanisms.

This study employed an integrative approach, which combined publicly available transcriptomic datasets, single-cell RNA sequencing, differential expression analysis, weighted gene co-expression network analysis (WGCNA), Venn diagram comparisons, machine learning algorithms, receiver operating characteristic (ROC) curve assessment, and experimental validation, to delineate macrophage-associated and mitophagy-related biomarkers in IPF, revealing novel regulatory networks. Further investigations utilized Gene Set Enrichment Analysis (GSEA), comprehensive immune infiltration profiling, and computational drug sensitivity prediction to delineate the molecular mechanisms governing the role of these biomarkers in the pathogenesis of IPF. Finally, single-cell transcriptomic data analysis enabled cell-type annotation within IPF samples and suggested the dynamic expression patterns of biomarkers during macrophage differentiation. These findings provide a robust foundation for deciphering IPF’s molecular mechanisms and developing novel therapeutic strategies. Future studies are expected to facilitate personalized and precise medical interventions tailored to individual patient profiles.

## 2. Results

### 2.1. Identification of DEGs1 in IPF

**Figure 1:**
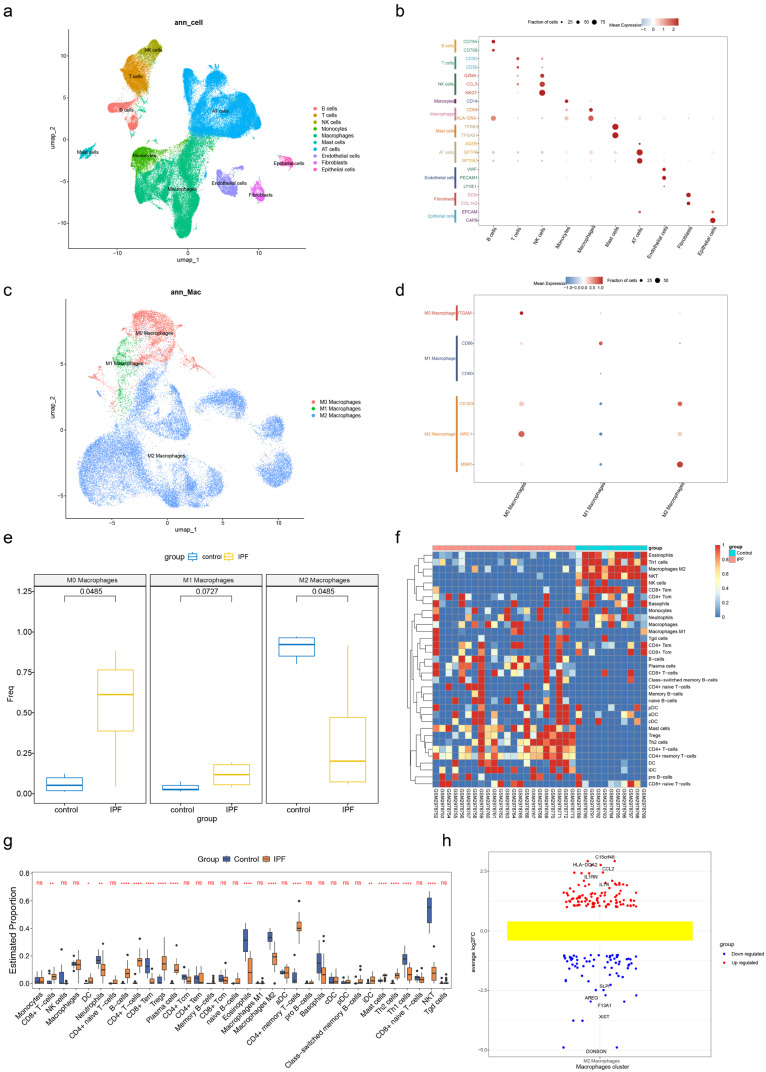
Identification of DEGs1 in IPF. (**a**) UMAP plot of major cell subgroups. Each point represents a cell, and different colors represent different subgroups of cells. (**b**) Bubble plot of cell subgroups and cell marker genes. The size of the circle represents the proportion of genes in the corresponding cell subgroup. Red: positive expression; blue: negative expression. (**c**) UMAP map of macrophage subgroups. Each point represents a cell, and different colors represent different subgroups of macrophages. (**d**) Bubble plot of macrophage subgroups and cell marker genes. The size of the circle represents the proportion of genes in the corresponding cell subgroup. Red: positive expression; blue: negative expression. (**e**) Differential expression map of macrophage subgroups between IPF patients and normal control samples. Yellow: IPF; blue: donor. *X*-axis: Three macrophage subtypes: M0 macrophages, M1 macrophages, M2 macrophages. *Y*-axis: Freq, representing the frequency or normalized expression level of the relevant genes. (**f**) Heat map of immune cell distribution in IPF patients and normal control samples. Blue: control; pink: IPF; red: positive correlation; blue: negative correlation. (**g**) Box plot for screening differential immune cells. Blue: control: orange: IPF. *X*-axis: Immune cell type *Y*-axis: Cell abundance percentage. ns: *p* ≥ 0.05, *: *p* < 0.05; **: *p* < 0.01; ****: *p* < 0.0001. (**h**) Differential gene map of core macrophage subgroups. Blue: down-regulated; red: up-regulated.

After filtering, the GSE122960 dataset, which was designated for cellular-level mechanistic exploration in this study, contained 102,270 cells and 23,429 genes (Supplementary Figure S1a). The data were standardized to extract 2000 HVGs (Supplementary Figure S1b), and 28 cell clusters were obtained after clustering analysis of the top 20 PCs (Supplementary Figure S1c–e). The 28 cell clusters were categorized into 10 distinct cell lineages, including endothelial cells, fibroblasts, mast cells, B cells, T cells, natural killer (NK) cells, monocytes, macrophages, alveolar type (AT) cells, and epithelial cells (Figure 1a). A bubble plot was created to visualize the expression profiles of marker genes in the 10 identified cell types (Figure 1b).

Subsequently, macrophage clusters were annotated into three macrophage subtypes: M0 macrophages, M1 macrophages, and M2 macrophages (Figure 1c). A bubble chart was created to visualize the expression of marker genes2 in the three macrophage subtypes, verifying the reliability of macrophage subtype annotation at the single-cell level (Figure 1d). After that, the Wilcoxon test demonstrated significant disparities in the abundance of M1 and M2 macrophages between IPF and control samples within the GSE122960 dataset (*p* = 0.0485) (Figure 1e), revealing the abnormal polarization pattern of macrophages in IPF at the cellular level and laying a single-cell foundation for subsequent subtype-centric analyses.

To complement single-cell resolution with bulk tissue-level transcriptomic characteristics, immune infiltration analysis of IPF patients uncovered significant divergences in 16 immune cell populations between IPF and control groups, including natural killer T (NKT) cells, T helper 1 (Th1) cells, central memory CD4+ T (CD4+ Tcm) cells, dendritic cells (DCs), CD4+ effector memory T (CD4+ Tem) cells, and regulatory T cells (Tregs) (*p* < 0.05). Among these cells, the infiltration levels of neutrophils, eosinophils, and M2 macrophages exhibited a notable decline in the IPF samples when compared with the control samples (Figure 1f,g), which was consistent with the macrophage abundance difference results in the single-cell clustering analysis, and cross-validated the abnormal immune cell infiltration pattern in IPF from both single-cell and bulk tissue dimensions. Consistently, based on these results, M2 macrophages were determined as the key immune subset linking cellular heterogeneity and tissue-level immune disorders, and were selected as the core macrophage subtype for subsequent analyses in this study. A total of 162 DEGs1 were found between IPF and control samples in M2 macrophages of the GSE122960 dataset. Among these, 98 DEGs1 were up-regulated and 64 were down-regulated (Figure 1h).

### 2.2. Identification and Functional Enrichment of DEGs2 in IPF

**Figure 2:**
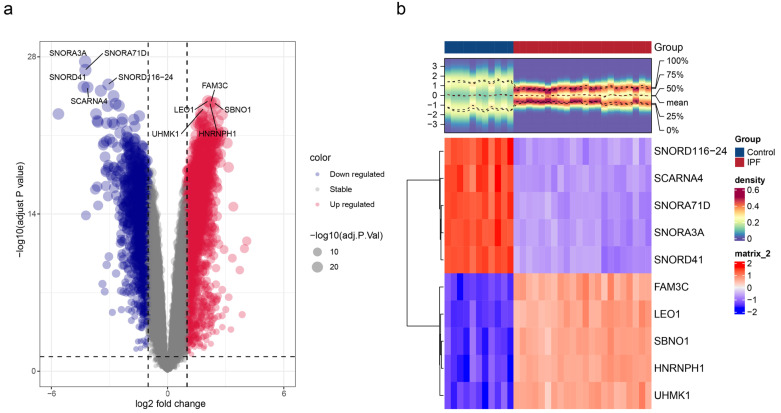
Identification of DEGs2 in IPF. (**a**) Volcanic map of differentially expressed gene distributions between IPF patients and normal control samples. The top 10 up- and down-regulated genes are shown. *X*-axis (log_2_ fold change, log_2_FC): Represents the expression fold change in molecules. A value farther from 0 indicates a more significant difference; positive values (right side) represent upregulation in the case group, while negative values (left side) represent downregulation, while negative values (left side) represent downregulation. *Y*-axis (−log_10_ (adj. *p* value)): Represents the statistical significance of differences. A higher value indicates a smaller *p* value and a more reliable result. (**b**) Heatmap of differential gene expression. Red: control groups; blue: IPF groups; red: positive correlation; blue: negative correlation.

To further capture global transcriptomic changes in IPF lung tissue and integrate with single-cell derived macrophage-related DEGs, a total of 3385 DEGs2 were identified between IPF and control samples within the GSE110147 dataset. Among them, 2110 DEGs2 were up-regulated and 1275 DEGs2 were down-regulated in IPF specimens. A volcano plot was used to label the top 5 up/down-regulated DEGs2, ranked by *p*.adjust (Figure 2a). A heat map was created to depict the mRNA expression of these genes (Figure 2b). The 3385 DEGs2 were involved in 527 GO terms, comprising 411 biological processes (BPs), 83 cellular components (CCs), 33 molecular functions (MFs), and 18 KEGG pathways (*p*.adjust < 0.05) (Supplementary Figure S2a,b, Supplementary Tables S2 and S3). These GO terms were regulation of mitotic sister chromatid segregation and ribonucleoprotein complex biogenesis in BP, ciliary plasm and axoneme in CC, ATP-dependent activity and acting on DNA, and helicase activity in MF. These KEGG pathways comprised amyotrophic lateral sclerosis, non-alcoholic fatty liver disease, the TNF signaling pathway, and other pathways.

### 2.3. Identification of 6992 MARMGs in IPF

**Figure 3:**
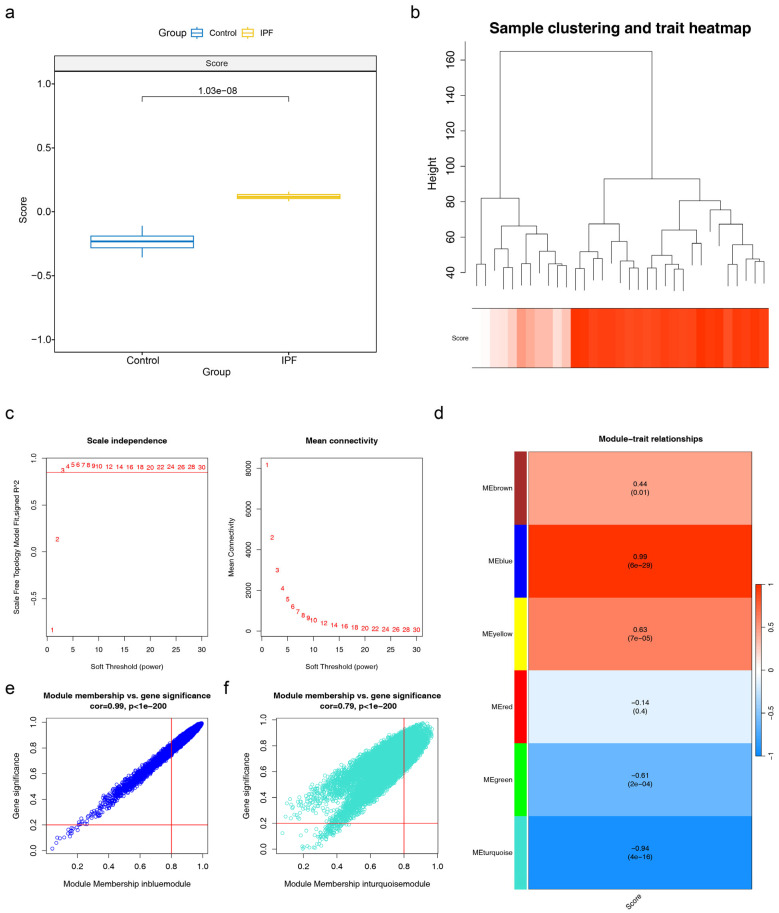
Identification of MARMGs in IPF. (**a**) Box plot of GSVA score comparison. Blue: control group; yellow: IPF group. (**b**) Box plot of CSPRG score comparison. Up: Sample distribution after clustering using the HCLUST function. Down: Cluster information of different samples. (**c**) Soft threshold filtering diagram. The value that crosses the red line of R^2^ = 0.9 is chosen as the soft threshold and its corresponding average connectivity is close to 0. (**d**) Heatmap of module and trait correlation. Red: positive correlation; blue: negative correlation. (**e**) Module genes of the MEblue module. (**f**) Scatter plot of GS and MM correlation strength for the key MEturquoise module. *X*-axis: MM: the correlation between genes and this module; *y*-axis: GS: the absolute value of the correlation between gene and trait scores. Red line: screening thresholds for GS and MM.

Given the critical role of mitophagy in macrophage polarization and lung fibrosis, the MARG score was significantly different in IPF and control samples within the GSE110147 dataset (*p* = 1.03 × 10^−8^) (Figure 3a). The clustering of WGCNA indicated the absence of any outlier samples in the GSE110147 dataset (Figure 3b). The β value was 14 based on an R^2^ of >0.9 and a mean connectivity of 0 (Figure 3c). The analysis identified six co-expression modules, among which the MEblue module demonstrated the strongest positive correlation with the MARG score (cor = 0.99, *p* = 6 × 10^−29^), while the MEturquoise module displayed the strongest inverse correlation with the MARG score (cor = −0.94, *p* = 4 × 10^−16^) (Figure 3d). The MEblue and MEturquoise modules contained 5264 and 1728 genes, respectively (Figure 3e,f); hence, they were recorded as potential key modules, featuring 6992 module genes that are MARMGs.

### 2.4. Identification of Two Biomarkers

**Figure 4:**
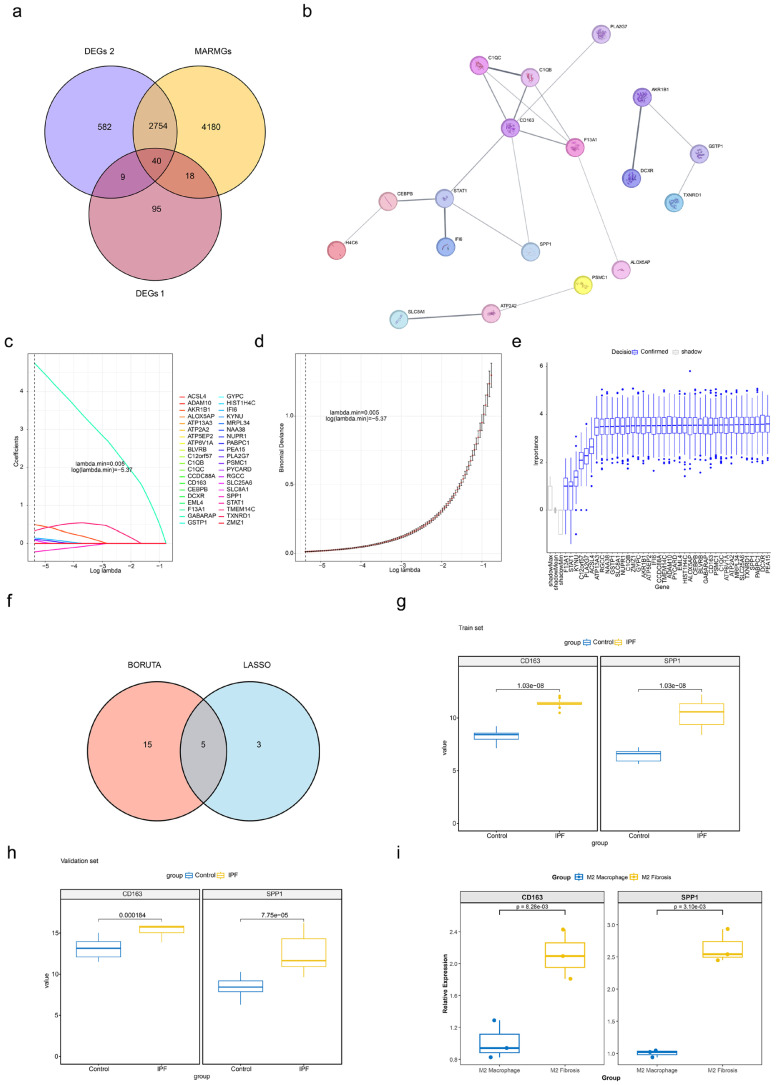
Identification of biomarkers. (**a**) Venn diagram of candidate genes. Forty candidate genes were obtained. (**b**) PPI network candidate genes. Each node represents a protein, and each edge represents the relationship between two proteins. The thickness of the edge indicates its importance in the network. (**c**) LASSO coefficient path diagram in the GSE110147 dataset. The dashed line represents the lambda value at which the error rate is minimized. *X*-axis: Number of features (number of genes). *Y*-axis: Model error. (**d**) LASSO cross-validation curve in the GSE110147 dataset. The dashed line represents the lambda value at which the error rate is minimized. *X*-axis: Log (λ), where λ is the regularization parameter. *Y*-axis: Gene regression coefficient. (**e**) BORUTA algorithm filtering results. The blue box represents the features filtered through validation, while the gray box represents the shadow features. (**f**) Venn diagram of five key genes. (**g**,**h**) Expression levels of featured genes in GSE110147 and GSE10667. Blue: control; yellow: IPF. (**i**) PCR validation of gene expression levels for candidate key genes.

To systematically integrate single-cell macrophage specificity, bulk tissue transcriptome changes, and mitophagy regulation, and identify potential IPF-related biomarkers, 40 candidate genes were pinpointed through the intersection of 162 DEGs1, 3385 DEGs2, and 6992 MARMGs (Figure 4a). The PPI network consisted of 39 nodes, 19 reciprocal pairs, and 21 isolated nodes, and example interactions include those between *CD163* and *F13A1* and *SPP1* and *STAT1* (Figure 4b).

LASSO regression analysis was performed using 5-fold cross-validation with lambda. min = 0.005. Eight genes with non-zero regression coefficients were identified (Figure 4c,d), and the Boruta algorithm identified 20 genes according to importance (Figure 4e). The five candidate genes (*SLC25A6*, *GABARAP*, *CD163*, *PEA15*, and *SPP1*) selected through LASSO and Boruta were taken as feature genes (Figure 4f).

The Wilcoxon test revealed significant discrepancies in *CD163* and *SPP1* expression between IPF and control specimens in both the GSE110147 and GSE10667 datasets (*p* < 0.05). Meanwhile, *CD163* and *SPP1* were notably up-regulated in the IPF samples (Figure 4g,h). The RT-qPCR results demonstrate that the model group exhibited higher expression of *SPP1* and *CD163* genes compared to the control group (Figure 4i). These results were consistently verified across multiple independent datasets and experimental validation, supporting the translational potential of *CD163* and *SPP1* as diagnostic or therapeutic markers. These results verified the reliable expression of *CD163* (used for M2 macrophage sorting and annotation) and *SPP1* in the study system, so *SPP1* and *CD163* were used as potential biomarkers linked to mitophagy-related pathways in IPF for subsequent analyses.

### 2.5. Biological Pathways, Immune Microenvironment, and Drugs for Two Biomarkers in IPF

**Figure 5:**
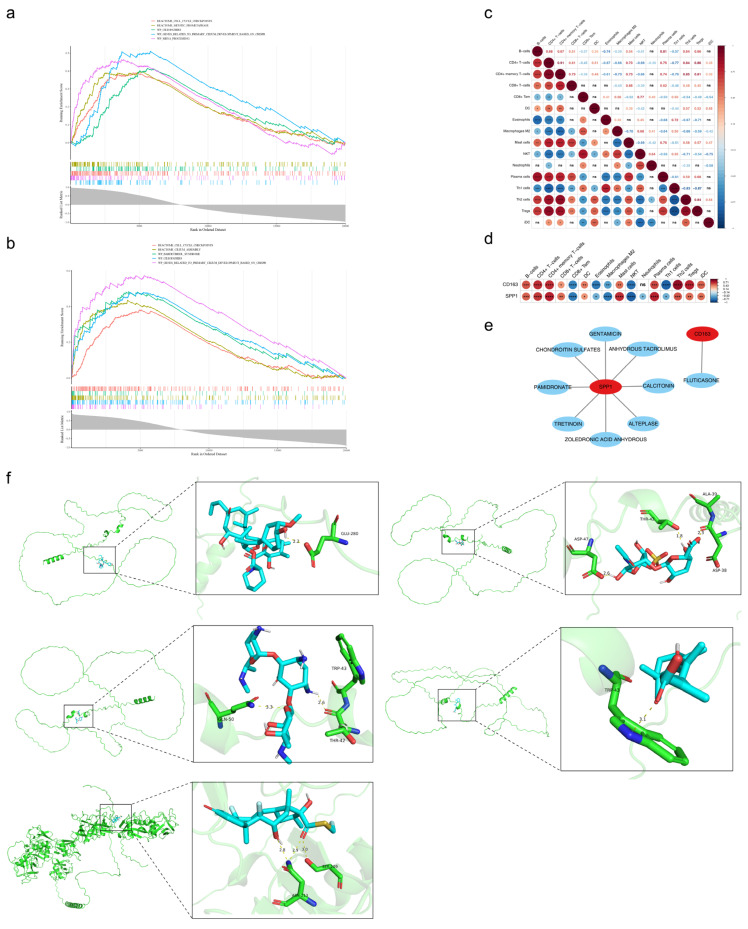
Biological pathways, immune microenvironment, and drugs for biomarkers in IPF. (**a**,**b**) GSEA of *CD-163* and *SPP1* biomarkers. The horizontal axis represents the ranking in the sorted gene set, the vertical axis represents the enrichment score, and different colored lines represent different pathways. (**c**) Heatmap of correlation analysis between differential immune cells. Red: positive correlation; blue: negative correlation. ns: *p* ≥ 0.05, *: *p* < 0.05; **: *p* < 0.01; ***: *p* < 0.001; ****: *p* < 0.0001. (**d**) Heat map of correlation analysis between biomarkers and differential immune cells. Red: positive correlation; blue: negative correlation. (**e**) Drug target network for biomarkers. Red: biomarker; blue: approved drugs. (**f**) Molecular docking between predicting drugs and key genes.

To explore the underlying mechanisms and translational value of the identified biomarkers, GSEA and immune correlation analyses were performed. In the GSEA, using the criteria of |NES| > 1, *p* < 0.05, and FDR < 0.25, it was found that *CD163* was significantly enriched in 318 pathways, including cell cycle checkpoints, ciliopathies, mRNA processing, GPCR ligand binding, and other pathways (Figure 5a, Supplementary Table S4). *SPP1* was significantly enriched in 272 pathways, including cell cycle checkpoints, ciliopathies, mitotic spindle checkpoint, separation of sister chromatids, and other pathways (Figure 5b, Supplementary Table S5). These findings indicate that *CD163* and *SPP1* jointly influence cell cycle checkpoints and ciliopathies, which may suggest a potential link to a shared pathological mechanism driving the development and progression of IPF.

The immune infiltration analysis of IPF patients uncovered a strikingly strong positive correlation between CD4+ memory T cells and CD4+ T cells (cor = 0.91, *p* < 0.001), whereas a markedly robust negative correlation was identified between Th1 cells and Tregs (cor = −0.87, *p* < 0.001) (Figure 5c). Moreover, a significant positive association was observed between *CD163* and T helper 2 cells (Th2 cells) (cor = 0.84, *p* < 0.001), while a significant negative association was found between *SPP1* and NKT cells (cor = 0.77, *p* < 0.001) (Figure 5d, Supplementary Table S6). These findings indicate that *CD163* may act synergistically with the infiltration level of Th2 cells to influence IPF.

To further expand the clinical translational value of this study, nine drugs associated with two biomarkers were predicted via DGIdb, including alteplase, calcitonin, gentamicin, chondroitin sulfates, tretinoin (associated with *SPP1*), and fluticasone (associated with *CD163*) (Figure 5e, Supplementary Table S7). These results suggest that *SPP1* and *CD163* may be potential targets for alteplase and fluticasone to treat IPF, providing candidate therapeutic strategies for future preclinical and clinical studies.

Molecular docking results showed that SPP1 displayed strong binding affinities for gentamicin, chondroitin sulfates, anhydrous tacrolimus, and tretinoin, exhibiting binding free energies of −4.9 kcal/mol, −5.0 kcal/mol, −5.8 kcal/mol, and −5.0 kcal/mol; CD163 displayed strong binding affinities for fluticasone, exhibiting binding free energies of −8.5 kcal/mol (Table 1). Notably, SPP1 shared 3 binding amino acid residues with gentamicin, including TRP-43, GLN-50, and THR-42; SPP1 shared 4 binding amino acid residues with chondroitin sulfate, including ASP-47, THR-42, ALA-39, and ASP-38; SPP1 shared 1 binding amino acid residue with anhydrous tacrolimus, including AGLU-280; SPP1 shared 1 binding amino acid residue with tretinoin, including TRP-43; CD163 shared 2 binding amino acid residues with fluticasone, including SER-209 and ASN-213 (Figure 5f). The findings suggested that SPP1 and CD163 interacted with these drugs, and such interactions might have therapeutic significance for patients with IPF.

### 2.6. Cellular Communication in IPF

**Figure 6:**
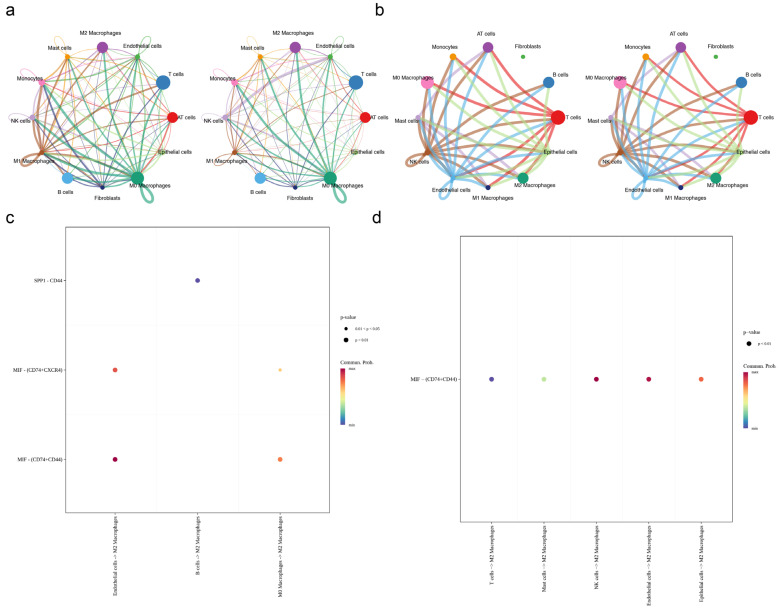
Cellular communication in IPF. (**a**) Left: the number of interactions between ligands and receptors in cellular subgroups in IPF; right: strength network of ligand–receptor interactions in cellular subgroups in IPF. Different colors represent different cell subgroups, and the thickness of the lines indicates the strength and quantity of interactions. The thicker the line, the stronger/more numerous the interactions. The arrows on the lines indicate the direction of the interactions. (**b**) Left: the number of interactions between ligands and receptors in cellular subgroups in the control; right: strength network of ligand–receptor interactions in cellular subgroups in the control. Different colors represent different cell subgroups, and the thickness of the lines indicates the strength and quantity of interactions. The thicker the line, the stronger/more numerous the interactions. The arrows on the lines indicate the direction of the interactions. (**c**) Bubble diagram of ligand–receptor interactions in IPF. Bubble color represents the communication probability (Commun. Prob.), with a color gradient from blue to red; red indicates a higher communication probability. Bubble size denotes the *p*-value, where larger bubbles correspond to smaller *p*-values and thus higher statistical significance. (**d**) Bubble diagram of ligand–receptor interactions in the control. Bubble color represents the communication probability (Commun. Prob.), with a color gradient from blue to red; red indicates a higher communication probability. Bubble size denotes the *p*-value, where larger bubbles correspond to smaller *p*-values and thus higher statistical significance.

To clarify how M2 macrophages and the two biomarkers function within the IPF cellular ecosystem, the findings of the cell communication analysis indicate that in IPF samples, M2 macrophages communicated more frequently with monocytes, while M2 macrophages and M0 macrophages interacted more frequently with endothelial cells and M2 macrophages, respectively. Notably, these cells demonstrated a greater intensity of communication with each other (Figure 6a). Conversely, in control samples, the interactions between epithelial cells and M2 macrophages, epithelial and B cells, epithelial and AT cells, epithelial cells and monocytes, and epithelial and endothelial cells were more conspicuous. Notably, these cells demonstrated a greater intensity of communication with each other (Figure 6b). The molecular results of intercellular communication signals show that, in IPF and control samples, the primary signals sent from endothelial cells to M2 macrophages were MIF− (CD74+ CD44) (Figure 6c,d). These findings demonstrate that M2 macrophages may interact with other cells to contribute to the pathogenesis of IPF, offering a cellular interaction basis for understanding the role of CD163 and SPP1 in the IPF microenvironment.

### 2.7. Pseudo-Temporal Trajectory of M2 Macrophages in IPF

**Figure 7:**
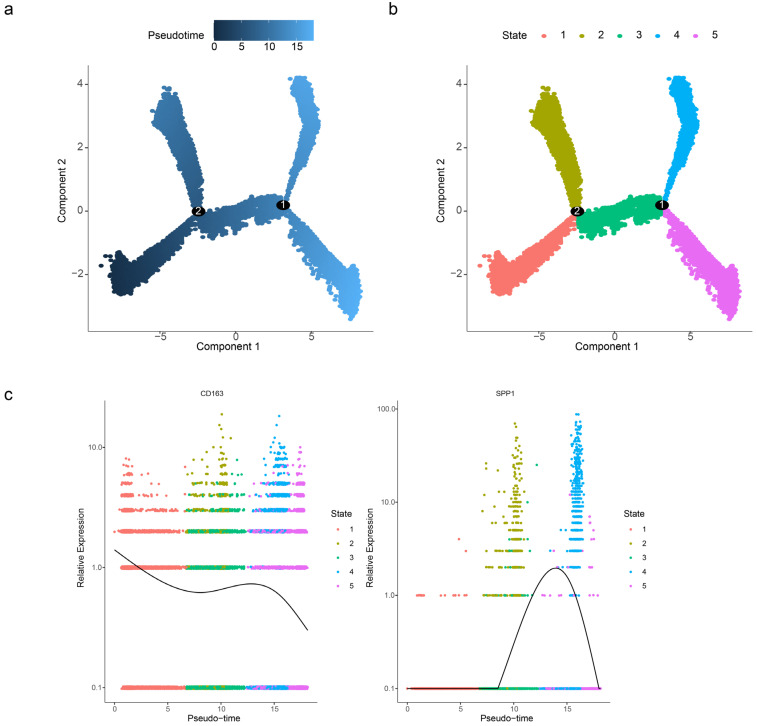
Pseudo-temporal trajectory of M2 macrophages in IPF. (**a**,**b**) Distribution map of core macrophages on differentiation trajectory. Axes: Component 1 and Component 2 represent the principal components after dimensionality reduction, which constitute the “map” of cell differentiation. Color coding: Represents pseudotime, with values ranging from 0 (dark blue) to 15 (light blue). Lower values indicate that cells are at the initial stage of differentiation, while higher values indicate the terminal stage. (**c**) Relationship diagram of key genes in core macrophages along pseudo-time. Different colors represent different differentiation states.

To characterize the dynamic behavior of M2 macrophages and the two biomarkers during IPF progression, pseudotime inference using the Monocle2 algorithm suggested a differentiation trajectory for M2 macrophages. Cells were ordered from left to right based on transcriptional similarity (Figure 7a,b). Along this inferred trajectory, CD163 expression exhibited a dynamic pattern. It initially decreased, then increased, and subsequently decreased again. SPP1 expression remained stable at first, followed by an increase and then a decrease (Figure 7c).

These results suggest that *CD163* and *SPP1* exhibit dynamic expression patterns during M2 macrophage differentiation, which may modulate macrophage functional transition and thus participate in the occurrence and development of IPF.

## 3. Discussion

In the pathological progression of IPF, cellular senescence and concomitant mitochondrial dysfunction play critical roles. Studies have demonstrated that defective mitophagy in senescent alveolar epithelial cells is closely associated with disease progression [12]. At the immunoregulatory level, phenotypic switching of macrophages (pro-inflammatory M1 versus anti-inflammatory M2) influences disease dynamics by modulating the fibrotic microenvironment [8]. Notably, alveolar macrophages in IPF patients exhibit significant mitochondrial homeostasis dysregulation [18], while Akt1 pathway-mediated mitophagy promotes fibrotic progression by enhancing apoptosis resistance [13]. Although clinically approved drugs pirfenidone and nintedanib can delay the decline in pulmonary function, they fail to reverse fibrosis and exhibit notable side effects [19]. This study systematically reveals the regulatory network linking macrophage polarization and mitophagy in IPF through bioinformatics screening combined with functional enrichment analysis, immune cell infiltration assessment, and single-cell sequencing, identifying *CD163* and *SPP1* as biomarkers. Experimental validation demonstrates significant differential expression of these biomarkers in an M2 macrophage fibrotic model, offering novel insights for clinical diagnosis and personalized therapeutic strategies in IPF patients.

*CD163*, a hemoglobin scavenger receptor exclusively expressed on macrophages, is a well-recognized canonical marker for identifying M2 macrophages and mediates the clearance of haptoglobin–hemoglobin complexes with anti-inflammatory properties by limiting oxidative tissue damage [20]. In the present study, CD163 was applied solely as a standard identifier for M2 macrophage annotation and sorting, and the related results were only used to verify the reliability of cell isolation, without any conclusion or implication regarding diagnostic value. The selective accumulation of *CD163*+ macrophages in pathological niches (e.g., chronic inflammation, tumors) has spurred interest in exploiting these cells for targeted drug delivery and leveraging their inherent tropism to minimize off-target toxicity [21]. In the pathological progression of IPF, pulmonary infiltration of *CD163*+ and *CD204*+ macrophages is significantly correlated with poor patient prognosis, suggesting that CD163+ macrophages participate in the pathogenesis of IPF by regulating macrophage activation states and the TGF-β1 secretion pathway, which may represent potential intervention strategies [22]. Serum analysis of IPF patients during acute exacerbation revealed abnormal upregulation of macrophage-related chemokines (s*CD163*, *CCL2*, *CXCL10*) and pro-inflammatory factors (particularly IFN-γ), which further support the functional involvement of CD163+ macrophages in IPF progression by activating macrophage functions [23]. In contrast to prior work that reported CD163+ macrophages in fibrosis as static entities [23], our single-cell trajectory analysis inferred dynamic co-expression of CD163 and SPP1 during macrophage differentiation (Figure 7c), proposing a novel mechanism where their synergy in the mitophagy network may drive IPF progression beyond classical paradigms. The pleiotropic cytokine *SPP1* (osteopontin) drives fibrosis via two mechanisms—(1) promoting M2 polarization through JAK2/STAT3-dependent transcriptional reprogramming and (2) amplifying PI3K/AKT signaling to enhance fibroblast activation and ECM deposition [24,25]—while simultaneously acting as a critical modulator of the PI3K/AKT pathway [26]. In vivo inhibition experiments confirm its effectiveness in delaying pulmonary fibrosis progression [27]. Notably, CD163+ macrophages identified in this study were the major cellular source of SPP1, indicating that CD163 and SPP1 synergistically contribute to the development of IPF by coordinating macrophage polarization and pro-fibrotic signaling. These findings together underscore the key functional potential of macrophage-derived CD163 and SPP1 in IPF, rather than their value as single biomarkers.

Based on molecular interaction network analysis and GSEA, it was revealed that *CD163* synergistically regulates fatty acid metabolism reprogramming and the *E2F* signaling pathway in coordination with *SPP1*. Macrophages are the most critical immune cells regulating the progression of IPF; they produce growth factors and mediators through various molecular mechanisms, such as immune receptors and cytokines, thereby driving the initiation and progression of IPF [28]. In bleomycin-induced mouse models of pulmonary fibrosis, alveolar macrophages (AMs) and monocyte-derived macrophages (mo-Macs) communicate with other immune cells through signals such as SPP1, CCL5, and CXCL2, exhibit pro-fibrotic gene expression heterogeneity and dynamic changes with disease progression, accompanied by upregulation of Gpnmb and Trem2 as well as metabolic remodeling, which further promotes pulmonary fibrosis [29]. The results demonstrate that dysregulated lipid metabolism and its involvement in immune regulation play pivotal regulatory roles in the pathological progression of IPF. In-depth exploration of the functional networks associated with fatty acid metabolism-related genes (FAMRGs) may unveil novel molecular targets for IPF therapy [30]. Notably, the APN/CPT1A signaling axis regulates lipid metabolism by enhancing autophagy activity, demonstrating significant protective effects in the lung and offering new insights for IPF intervention strategies [31]. Furthermore, targeted modulation of the E2F2/HO-1 signaling pathway has exhibited therapeutic potential in animal models of pulmonary fibrosis, highlighting its translational value for fibrotic disease treatment [32].

The results of the analysis of the immune microenvironment further clarify the functional roles of *CD163* and *SPP1* in IPF. The abnormal accumulation of NKT cells, Th1 cells, and dendritic cells (DCs) in the lung tissues of IPF patients indicates dysregulation of immune homeostasis. Studies demonstrate that DCs may induce Th2-type immune responses through autoantigen presentation, thereby promoting M2 macrophage polarization [33]. Concurrently, aberrant activation of invariant natural killer T (iNKT) cells can trigger neutrophil infiltration and activate inflammasomes, macrophages, and fibroblasts, ultimately leading to fibrotic lesion formation [34]. Notably, the expression levels of *CD163* and *SPP1* are positively correlated with the infiltration of CD4+ T cells and their memory subsets, suggesting their potential involvement in immune tolerance imbalance through the regulation of T-cell subset differentiation. Consequently, targeting early signaling pathways of these immune responses (e.g., inhibiting iNKT cell activation) may represent a critical strategy for modulating fibrotic progression [34]. Research confirms that *SPP1*-high macrophages play a central role in pulmonary fibrosis, while intrapulmonary accumulation of *CD163*+ macrophages is associated with a poor prognosis in IPF patients. Therapeutic strategies targeting the MERTK pathway, suppressing macrophage proliferation/activation, and blocking TGF-β1 secretion have demonstrated potential clinical value [22,34].

Our study revealed that a total of nine drugs are associated with these two biomarkers, including alteplase, calcitonin, gentamicin, chondroitin sulfate, and retinoic acid, which are associated with *SPP1*, and fluticasone, which is associated with *CD163*. The drug targeting strategies identified in this study show significant correlations with previous research.

Regarding *SPP1*-associated drugs, the mechanism of action of alteplase is highly consistent with that reported by Yang Miao et al. [35]. Their study confirmed that activation of the fibrinolytic system can significantly alleviate the progression of pulmonary fibrosis by modulating the *SPP1*/integrin signaling pathway. For *CD163*-associated drugs, our results corroborate the findings of Florence Vallelian’s team [36]. Their research demonstrated that glucocorticoids up-regulate *CD163* expression to promote the clearance of hemoglobin–haptoglobin complexes, thereby reducing pulmonary oxidative stress injury. Particularly noteworthy is the recent study by Zhong et al. [2], which uncovered the core regulatory role of the *Clec7a/NF-κB/SPP1* signaling axis in pulmonary fibrosis. This finding provides a mechanistic complement to our discoveries. Furthermore, during pulmonary fibrosis, abnormal sulfation modifications of chondroitin sulfate/dermatan sulfate (CS/DS) occur alongside up-regulation of glucuronosyltransferase-I (GlcAT-I) expression. Together, these promote the pathological accumulation of proteoglycan–glycosaminoglycan complexes [37]. Collectively, this evidence indicates that although the drugs predicted in this study target different sites, they exhibit significant synergistic effects with known anti-fibrotic mechanisms. This provides crucial theoretical support for developing multi-target combination therapies based on the *SPP1-CD163* signaling network.

Clinical studies have revealed that the levels of *CD163*+ and *CD204*+ cell infiltration in lung tissue are significantly associated with a poor prognosis in IPF patients. Research indicates that abnormal accumulation of M2 macrophages in the lungs may contribute to fibrotic progression by upregulating TGF-β1 expression, suggesting that the activation status of these cells and their secreted transforming growth factors could serve as critical therapeutic targets for fibrosis intervention [22]. Mechanistic investigations demonstrate that the *SPP1* molecule promotes macrophage polarization toward the M2 phenotype via activation of the *JAK2/STAT3* pathway, while animal models confirm that blocking *SPP1* expression effectively delays the pathological progression of pulmonary fibrosis [38]. Experimental data show that inhibition of Clec7a disrupts NFκB signaling-mediated *SPP1* secretion in M2 macrophages, with such intervention markedly improving tissue fibrosis in bleomycin-induced pulmonary fibrosis models. Notably, the antifibrotic drug nintedanib may exert therapeutic effects by targeting Clec7a in interstitial macrophages, thereby interfering with the *NFκ*B/*SPP1* signaling cascade.

In this study, biomarkers CD163 and SPP1 associated with macrophage–mitophagy were identified in idiopathic pulmonary fibrosis (IPF) based on public databases. Their functions, immune roles, and therapeutic potential were explored, which provided a theoretical basis and potential targets for the early diagnosis and precise treatment of IPF. However, several limitations were noted in the present study. First, the bioinformatics analyses relied on bulk RNA sequencing data generated from different platforms, which might have affected the accuracy of the results. Second, experimental validation was performed only in the THP-1 cell line and an in vitro two-dimensional model, which exhibited considerable differences from the in vivo microenvironment; meanwhile, verification at the protein level was lacking. And, CD163/SPP1 was associated with mitochondrial autophagy, but functional evidence (such as transmission electron microscopy (TEM) for visualization of mitochondrial autophagosomes or LC3-II/I ratio analysis) was lacking. Future studies will include gene knockout experiments in macrophages and combine mitochondrial function assays to establish causality. Third, the sample size was limited, and patients with diverse clinical characteristics and different disease stages were not included. In future work, validation at the protein level and morphological analyses will be supplemented, and molecular mechanisms will be further clarified through genetic manipulation. The sample size will be expanded, and multiple bioinformatic methods will be integrated to reduce the impact of heterogeneity. Multi-omics techniques will be employed to analyze molecular dynamics at different disease stages, and the findings will be validated in clinical samples and three-dimensional models to improve the rigor of this study.

## 4. Materials and Methods

### 4.1. Data Acquisition

Gene expression profiles of IPF-related datasets GSE110147, GSE10667 and GSE122960 were obtained from the Gene Expression Omnibus (GEO, http://www.ncbi.nlm.nih.gov/geo/, accessed on 1 Febraury 2025). A differential analysis design was adopted for the three datasets in this study: GSE110147, which has the advantages of a large sample size and well-defined clinical phenotypes making it suitable for reliable differential expression analysis, was used as the primary dataset to screen biomarkers and explore their mechanisms of action in IPF, GSE10667 was mainly used to verify the expression levels of the identified biomarkers, and GSE122960 was applied to investigate their cellular-level functions. Specifically, the GSE110147 dataset (platform: GPL6244) comprised lung tissue samples from 22 IPF patients and 11 healthy controls [39]; the GSE10667 dataset (platform: GPL4133) included lung tissue samples from 8 IPF patients and 15 healthy controls [40]; and the GSE122960 scRNA-seq dataset (platform: GPL20301) contained lung tissue samples from 5 IPF patients and 8 healthy controls [41]. Additionally, 71 mitophagy-associated genes (MARGs) were curated from the Molecular Signature Database (MSigDB) repository (https://www.gsea-msigdb.org/gsea/msigdb/human/geneset/GOBP_MITOPHAGY.html, accessed on 1 Febraury 2025) (Supplementary Table S1).

### 4.2. scRNA-seq Analysis

The GSE122960 dataset was analyzed using the Seurat (V5.1.0) package [42]. First, high-quality cells/genes were selected (percent.mt < 15%, 200 ≤ nFeature_RNA ≤ 4000, nCount_RNA < 10,000), and genes detected in <3 cells were filtered out. Next, filtered single-cell data were normalized via NormalizeData; highly variable genes (HVGs) were identified using FindVariableFeatures, with variability results visualized via VariableFeaturePlot. Then, HVGs were scaled via ScaleData, and principal component analysis (PCA) (JackStraw algorithm, *p* < 0.05) was performed on normalized data. Statistically significant principal components (PCs) were selected for downstream analyses, with dimensionality reduction results visualized via ElbowPlot. Finally, unsupervised clustering (FindNeighbors/FindClusters, resolution = 0.75) identified cell clusters, and results were visualized via uniform manifold approximation and projection (UMAP) (DimPlot function).

To characterize the cell clusters into distinct cell types, marker genes from a published reference [41] were utilized to annotate all clusters, with the DimPlot function employed to visualize the annotation outcomes. A bubble plot was generated to illustrate the expression profiles of marker genes across different cell types.

Following this, to further analyze the macrophages and identify differentiated macrophage subgroups, macrophages were selected from the scRNA-seq sequencing data. Unsupervised cluster analysis was performed on the downscaled data using statistically significant PCs from PCA, with the help of the FindClusters and FindNeighbors functions (resolution = 0.75), to identify different macrophage clusters. For cell type annotation, we employed established marker genes for M2 macrophage identification, as consistently used in the field. Subsequently, our analysis focused on identifying differentially expressed genes within these pre-defined M2 macrophages [43]. Next, the Wilcoxon test was used to compare the dissimilarities in the abundance of each macrophage subtype between diverse groups (IPF vs. control) in the scRNA-seq dataset (*p* < 0.05).

### 4.3. Identification of Differentially Expressed genes1 (DEGs1) in Core Macrophage Subtypes

To obtain the core macrophage subtypes, first, the relative proportions of the distribution of 34 immune cell types [44] were calculated using the xCell algorithm in IPF patients and normal control samples within the GSE110147 dataset. After that, the Wilcoxon test was used to select immune cells with marked discrepancies in a comparison of the two groups (*p* < 0.05). Macrophage subsets exhibiting statistically significant differences between IPF and control samples in both GSE110147 and GSE122960 datasets were designated as core macrophage subtypes for downstream analyses.

In the IPF and control groups of the GSE122960 dataset, the FindMarkers function was used to identify DEGs1 at the cellular level in core macrophage subtypes (|average log2 foldchange (FC)| ≥ 1, *p*.adjust < 0.05). The Manhattan plot depicting DEGs1 was generated using the ggplot2 package (V 3.5.1) [45].

### 4.4. Identification and Functional Analysis of Differentially Expressed genes2 (DEGs2)

The limma (V 3.58.1) package [46] was utilized to identify DEGs2 between IPF and control samples in the GSE110147 dataset, with significance defined as *p*.adjust < 0.05 and |log2 FoldChange (FC)| > 1. The ggplot2 package (V 3.5.1) was leveraged to craft a volcano plot depicting DEGs2. A heatmap was generated by making use of the ComplexHeatmap (V 2.21.1) package [47]. Only the top 5 up- and down-regulated genes (ranked in ascending order by *p*.adjust value) were displayed, respectively.

To evaluate the functions associated with the DEGs2, the clusterProfiler (V 4.8.3) package [48] was used to conduct Gene Ontology (GO) and Kyoto Encyclopedia of Genes and Genomes (KEGG) enrichment analysis on DEGs2 (*p*.adjust < 0.05).

### 4.5. WGCNA

Leveraging the plage algorithm from the GSVA (V 1.50.0) package [49], we calculated MARG scores for IPF and control samples from GSE110147. Group comparisons were performed via the Wilcoxon test (*p* < 0.05).

Subsequently, WGCNA was executed by means of the WGCNA (V 1.73) package [50]. First, the goodSamplesGenes function was used to remove ineligible genes and samples and the hclust function was used to cluster the samples. To obtain a suitable soft threshold (β) for network topology analysis, the pickSoftThreshold function was used to compute the β power and the scale-free fitting exponent to realize a scale-free topology. β was set according to the scale-free fitting index (R^2^), which was greater than 0.9, and the average connectivity, which was close to zero.

Briefly, a filtered expression matrix was utilized to construct the WGCNA network. The minimum module size was set at 35, the merge cut height at 0.3, and the module detection sensitivity at 2. With these parameters, co-expression modules were identified, and a hierarchical clustering tree was generated. Using MARG scores as phenotypic traits, the correlation matrix between MARG scores and modules was calculated via Pearson’s correlation (|cor| > 0.30, *p* < 0.05). Modules showing the strongest positive and negative correlations were defined as key modules. Module Membership (MM) and Gene Significance (GS) were then analyzed, and genes with |MM| > 0.8 and |GS| > 0.2 were identified as MARMGs, suggesting potential regulatory relationships.

### 4.6. Identification of Biomarkers

The ggvenn (V 0.1.10) package (https://CRAN.R-project.org/package=ggvenn, accessed on 20 February 2025) was used to identify the intersection of differentially expressed genes (DEGs1), DEGs2 and MARMGs, and the overlapping genes were defined as candidate genes.

To explore the functional interaction characteristics of candidate genes, the Search Tool for the Retrieval of Interacting Genes (STRING) database (http://www.string-db.org/, accessed on 21 February 2025) was employed to construct the protein–protein interaction (PPI) network of proteins encoded by candidate genes, with the confidence score threshold set at >0.4. The PPI network was then visualized using Cytoscape (V 3.10.3) software [51] for preliminary functional annotation and interaction pattern analysis of candidate genes.

For the precise screening of feature genes, machine learning approaches were sequentially applied to the candidate genes from the GSE110147 dataset: (1) Least absolute shrinkage and selection operator (LASSO) logistic regression analysis was performed using the glmnet (V 4.1.8) package [52], with 5-fold cross-validation for model validation to screen genes with strong predictive ability; (2) The Boruta algorithm was used for feature selection via the Boruta (V 8.0.0) package [53] to identify genes with significant discriminatory power. Subsequently, the ggvenn (V 0.1.10) package was utilized to obtain the gene intersection of the two machine learning results, and these overlapping genes were designated as feature genes.

To characterize the expression profiles of feature genes and verify their differential expression specificity, Wilcoxon test (*p* < 0.05) was performed to compare the expression levels of feature genes between IPF and control samples in both the GSE110147 and GSE10667 datasets. Finally, biomarkers were screened based on the following two criteria: (1) significant differential expression between groups in both datasets after FDR correction; (2) consistent expression trends across the two independent datasets.

### 4.7. GSEA and Immune Microenvironment Analysis

To explore the biological functions of biomarkers during IPF progression, the psych (V 2.4.3) package (https://CRAN.R-project.org/package=psych, accessed on 22 February 2025) was used to calculate Spearman correlations between biomarkers and other genes in the GSE110147 dataset. Correlation coefficients were then ranked in descending order. GSEA was conducted with the clusterProfiler (V 4.8.3) package, using the gene set c2.cp.all.v2022.1.Hs.symbols.gmt from the GSEA database (https://www.gsea-msigdb.org/gsea/index.jsp, accessed on 22 February 2025). Thresholds were set as |normalized enrichment score (NES)| > 1, *p* < 0.05, and false discovery rate (FDR) < 0.25. The top 5 significant pathways were selected for visualization.

To further explore the inter-relationships among differential immune cells and the associations between biomarkers and these cells, the cor function was utilized to calculate the Spearman correlation across all samples in the GSE110147 dataset. The aim of this was to illustrate the relationships among the differential immune cells and between the differential immune cells and biomarkers, with absolute correlation coefficients exceeding 0.3 and *p*-values < 0.05.

### 4.8. Drug Predictive Analysis

To discover biomarker-linked therapeutic agents, the Drug–Gene Interaction Database (DGIdb) (https://www.dgidb.org, accessed on 25 February 2025) was leveraged to predict drugs that are associated with the identified biomarkers. After that, the relationship between drugs and biomarkers was visualized using Cytoscape (V 3.10.3) software.

To verify the binding performance between the predicted candidates and key genes, drug structures were first downloaded from the PubChem database (https://pubchem.ncbi.nlm.nih.gov/, accessed on 22 March 2026) (added during the second revision), and protein structures encoded by the key genes were retrieved from the UniProt database (https://www.uniprot.org/, accessed on 22 March 2026) (added during the second revision). Subsequently, molecular docking between the drugs and corresponding proteins was performed using the CB-DOCK2 software (https://cadd.labshare.cn/cb-dock2/, accessed on 22 March 2026) (added during the second revision). Finally, the folding patterns and molecular interactions between proteins and drugs were analyzed, and the binding energies were obtained.

### 4.9. Cellular Communication and Pseudo-Temporal Trajectory Analyses

The CellChat (V 1.6.1) package [54] was adopted to investigate the interactions between key cells as well as other annotated cell types in IPF and control samples. Bubble plots were employed to illustrate the interactions between ligands and receptors in the core macrophage subtype within both IPF and control specimens (*p* < 0.05).

To dissect transcriptional expression of known biomarkers across core macrophage polarization states, dimensionality reduction clustering was performed on scRNA-seq data via the reduceDimension and DDRTree functions of the Monocle2 (V 2.28.0) package [55] (max_components = 2). Core macrophages were then sorted by biomarker expression, with pseudo-time values assigned using the orderCells function. Core macrophage subtypes were projected into a root and two branches to infer single-cell pseudo-temporal trajectories by biomarker expression patterns. After re-sorting cells via orderCells, subtypes were classified and visualized with plot_cell_trajectory; finally, biomarker expression trends during subtype development were analyzed using the Monocle2 plot_genes_in_pseudotime function.

### 4.10. Reverse Transcription Quantitative Polymerase Chain Reaction (RT-qPCR)

Human monocytic leukemia THP-1 cells (Suzhou Haixing Biosciences) underwent M0 macrophage polarization via 24-h exposure to 150 ng/mL phorbol 12-myristate 13-acetate (PMA). According to laws and regulations, as well as the relevant regulations of the institution, there was no need to obtain approval from the ethics committee, as the cell lines involved in this study were all commercial products. The experimental groups were as follows: (1) M2 group—M0 macrophages polarized with 30 ng/mL interleukin-4 (IL-4) for 48 h, and (2) the M2 fibrotic subgroup was established by further stimulating IL-4-induced M2 macrophages with 20 ng/mL transforming growth factor-β1 (TGF-β1) for 48 h to construct an in vitro M2 fibrotic macrophage model. Total RNA was isolated using the RNAeasy™ Animal RNA Isolation Kit, followed by genomic DNA elimination with gDNA Eraser and reverse transcription to cDNA. Target gene expression was quantified by RT-qPCR using the comparative ΔΔCq method (2−ΔΔCq), with GAPDH as the endogenous reference (F:5′-ACAACTTTGGTATCGTGGAAGG-3′; R:5′-GCCATCACGCCACAGTTTC-3′). Gene-specific primers included the following: *SPP1* (F:5′-GAAGTTTCGCAGACCTGACAT-3′; R:5′-GTATGCACCATTCAACTCCTCG-3′) *CD163,* a known specific marker of M2 macrophages, (F:5′-GACGCATTTGGATGGATCATGT-3′; R:5′-GACGCATTTGGATGGATCATGT-3′) was used for the identification and validation of M2 macrophage polarization.

### 4.11. Statistical Analysis

Data were processed and analyzed in R software (V 4.3.1) and group comparisons were conducted using the Wilcoxon test. Unless otherwise stated, statistical significance was defined as a *p*-value < 0.05.

## 5. Conclusions

Our integrated analysis revealed the upregulation of CD163 and SPP1 in the macrophage–mitochondrial autophagy network, providing more refined insights into the pathogenesis of IPF and thereby supplementing existing knowledge. A strong positive correlation was observed between Th2 cells and CD163. Furthermore, dynamic expression of the two genes was detected during M2 macrophage differentiation. The results suggest a potential association between SPP1/CD163 expression and mitophagy-related pathways, based on co-expression patterns and enrichment results. This generates a hypothesis for future mechanistic studies, but it does not establish causal synergy or clarify biological roles in a mechanistic sense.

## Figures and Tables

**Table 1:** The binding affinity between predicting drugs and key genes.

Gene (Receptor)	Gene Source	Drug (Ligand)	Drug CID	Vina Score (kcal/mol)
SPP1	AlphaFold AF-P10451-5-F1	GENTAMICIN	3467	−4.9
SPP1	AlphaFold AF-P10451-5-F1	CHONDROITIN SULFATES	24,766	−5.0
SPP1	AlphaFold AF-P10451-5-F1	ANHYDROUS TACROLIMUS	445,643	−5.8
SPP1	AlphaFold AF-P10451-5-F1	TRETINOIN	444,795	−5.0
CD163	AlphaFold AF-Q86VB7-2-F1	FLUTICASONE	5,311,101	−8.5

## Data Availability

The datasets analyzed in this study are publicly available: the training (GSE110147), validation (GSE10667), and single-cell (GSE122960) datasets were from GEO; mitophagy-related genes were from MSigDB (GOBP_MITOPHAGY). Processed data are provided in Supplementary Materials, and analysis scripts are available upon request.

## Supplementary Materials

The following supporting information can be downloaded at: https://www.mdpi.com/article/10.3390/ijms27104201/s1.

## Abbreviations

The following abbreviations are used in this manuscript:

IPF	Idiopathic Pulmonary Fibrosis
scRNA-seq	Single-Cell RNA Sequencing
DEGs1	Differentially Expressed Genes 1
WGCNA	Weighted Gene Co-Expression Network Analysis
ROC	Receiver Operating Characteristic
Th2 cells	T Helper 2 Cells
ECM	Excessive Extracellular Matrix
GSEA	Gene Set Enrichment Analysis
GEO	Gene Expression Omnibus
MARGs	Mitophagy-Associated Genes
MSigDB	Molecular Signature Database
HVGs	Highly Variable Genes
PCA	Principal Component Analysis
PCs	Principal Components
UMAP	Uniform Manifold Approximation and Projection
FC	Foldchange
DEGs2	Differentially Expressed Genes2
GO	Gene Ontology
KEGG	Kyoto Encyclopedia of Genes and Genomes
MM	Module Membership
GS	Gene Significance
MARMGs	Mitophagy-Related Module Genes
PPI	Protein–Protein Interaction
STRING	Search Tool for the Retrieval of Interacting Genes
LASSO	Least Absolute Shrinkage and Selection Operator
AUC	Area Under the Curve
NES	Normalized Enrichment Score
FDR	False Discovery Rate
DGIdb	Drug–Gene Interaction Database
RT-qPCR	Reverse Transcription Quantitative Polymerase Chain Reaction
IL-4	Interleukin-4
