# Supplementary figures and images for: Integrated Single-Cell and Bulk RNA Sequencing Identifies Macrophage Heterogeneity and Mitophagy-Related Biomarkers in Idiopathic Pulmonary Fibrosis
Source: Int J Mol Sci. 2026 May 8;27(10):4201. doi: 10.3390/ijms27104201 (PMC13208039; doi:10.3390/ijms27104201)

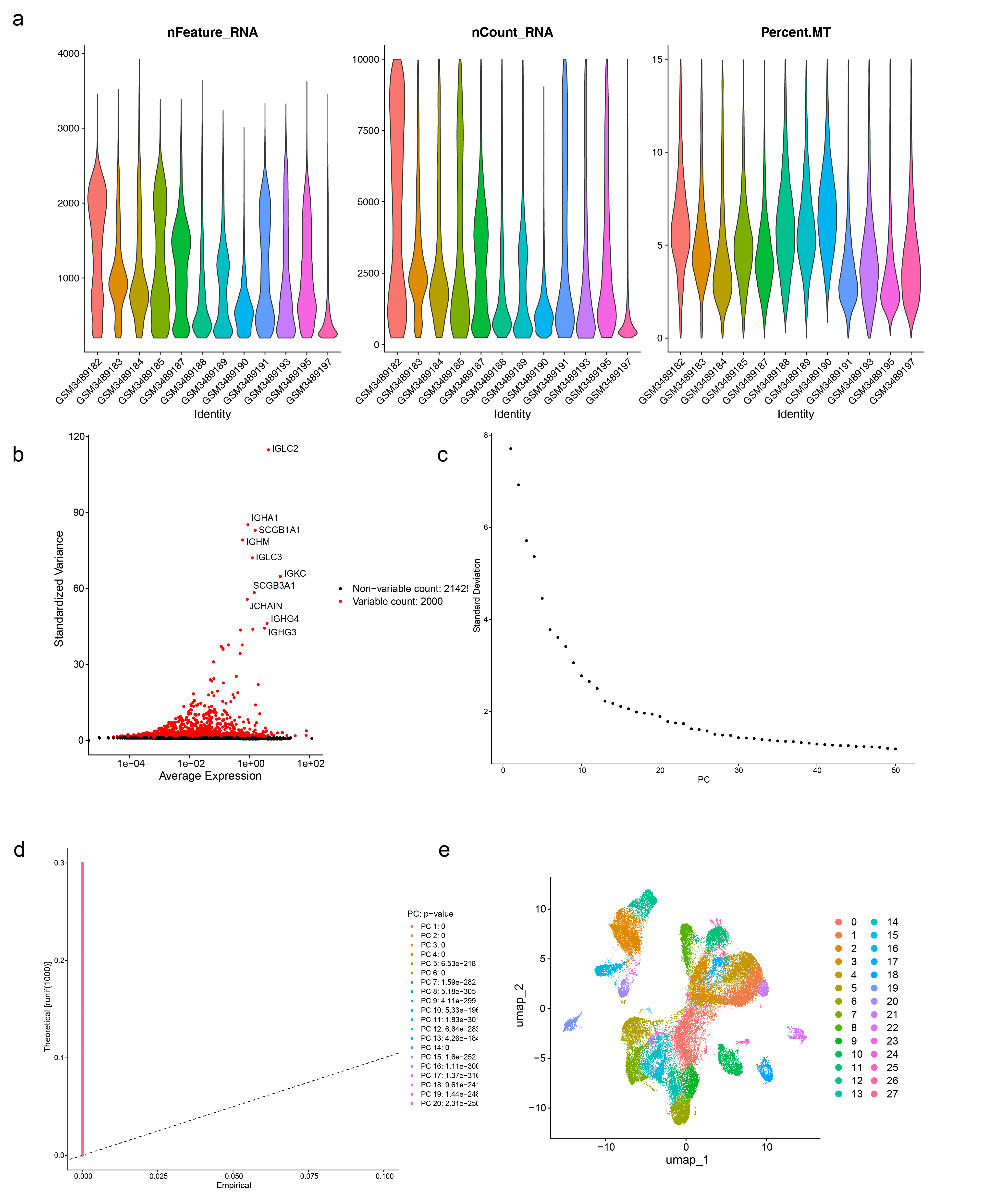

Supplement: Supplementary file 1 [file ijms-27-04201-s001.zip › Supplementary materials/Supplementary Figure S1.tif]

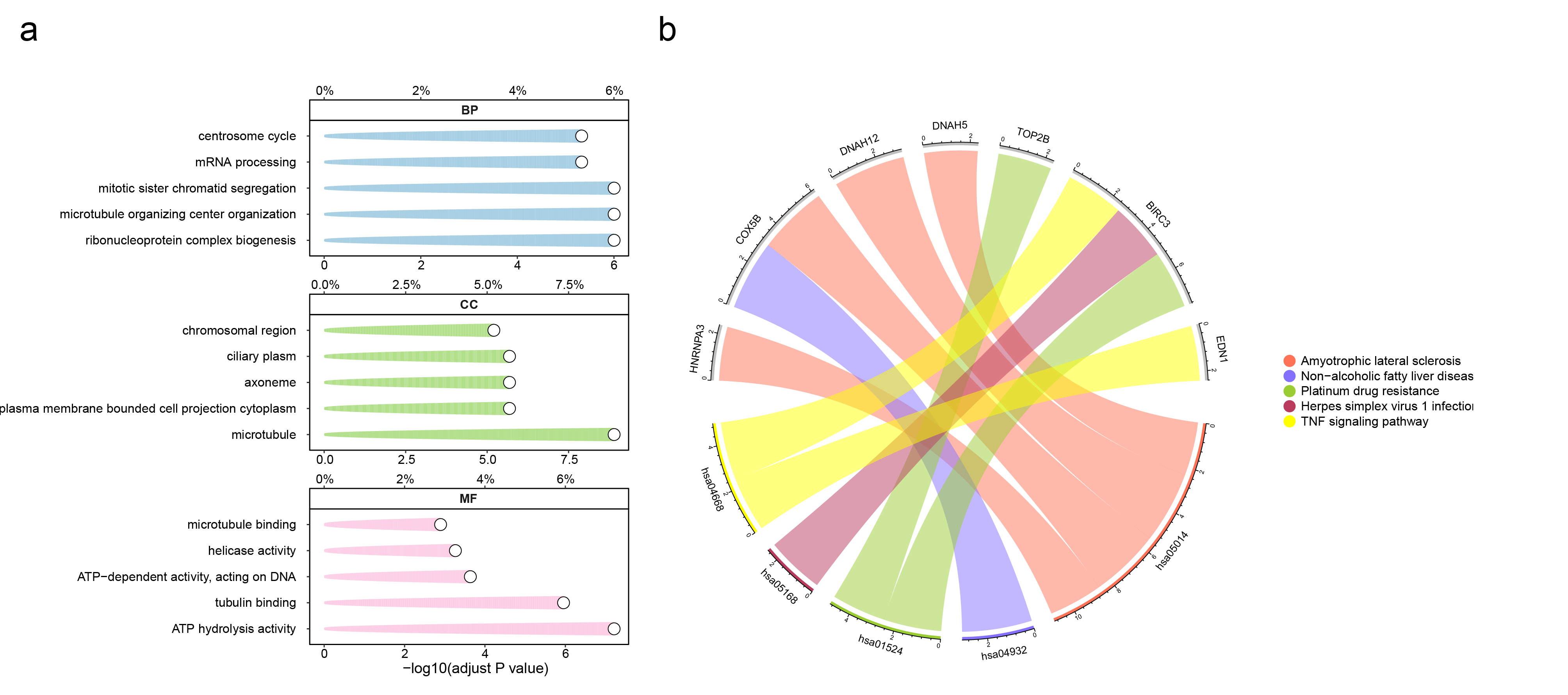

Supplement: Supplementary file 1 [file ijms-27-04201-s001.zip › Supplementary materials/Supplementary Figure S2.tif]
